# Functional outcomes and complications of classic grammont-style reverse shoulder arthroplasty in patients with os acromiale: a retrospective case-control study

**DOI:** 10.1007/s00264-025-06596-z

**Published:** 2025-07-02

**Authors:** Yaiza Lopiz, Raul Herzog, Camilla Arvinius, Carlos Garcia, Esperanza Anhui, Fernando Marco

**Affiliations:** 1 Shoulder and Elbow Unit. Department of Traumatology and Orthopaedic Surgery. Clínico San Carlos Hospital., Madrid, Spain; 2 Department of Surgery. Complutense University., Madrid, Spain

**Keywords:** Os acromiale, Reverse total shoulder arthroplasty, Cuff tear arthropathy, Constant score, Shoulder pain, Radiographs

## Abstract

**Purpose:**

To determine the functional impact and complications associated with os acromiale after the implantation of a reverse total shoulder arthroplasty (RTSA) with medialization of the centre of rotation.

**Methods:**

A retrospective case-control study with cross-sectional evaluation was conducted. Between 2004 and 2021, patients who underwent RTSA for cuff arthropathy, GH osteoarthritis or massive irreparable rotator cuff tears with os acromiale (OA) and more thantwo years of follow-up, were identified. A control cohort (2:1) without acromial compromise (NOOA) was also identified. Functional (Constant, ASES, Quick-DASH, VAS, ROM) and radiological assessment (os acromiale type, acromiohumeral distance, acromion tilt) were performed.

**Results:**

RTSA was implanted in 432 cases during the study period, 221 with rotator cuff arthropathy, irreparable tears, or osteoarthritis, of these, 12 had an os acromiale (OA) (5.4%) and were compared to 24 patients without os acromiale (NOOA). Epidemiologic data OA/NOOA were: female 10/20, mean follow-up 47.2 ± 25/56.1 ± 30 months, mean age 73.5 ± 4.7/75.4 ± 4.1 years. Regarding the difference in preoperative and final follow-up functional outcomes (OA/NOOA): Constant 20.2/30.9 (*p* =.012), ASES 28/54 (*p* =.017), Quick-DASH − 19.6/-27.2 (*p* =.220), VAS − 5/-7 (*p* =.007), difference in pre-surgery/last follow-up ROM: elevation 50º/60º (*p* =.138), abduction 60º/60º (*p* =.775). The os acromiale group presented two prosthetic dislocations (16.7%).

**Conclusion:**

Patients with os acromiale improve their preoperative condition after RTSA implantation; however, although there are no differences in joint balance, this improvement is significantly lower in the Constant and ASES scores, primarily due to a decrease in strength and pain relief experienced by patients with os acromiale.

## Introduction

Grammont designed a reverse total shoulder arthroplasty (RTSA) with a medialized and distalized centre of rotation, which increased the deltoid lever arm to prevent early loosening of the glenosphere. This has been reported to produce satisfactory early clinical outcomes [1]; however, there have also been concerns regarding arm lengthening and complications such as acromion or spine fractures which has been related to poor functional results [2, 3].

**Fig. 1:**
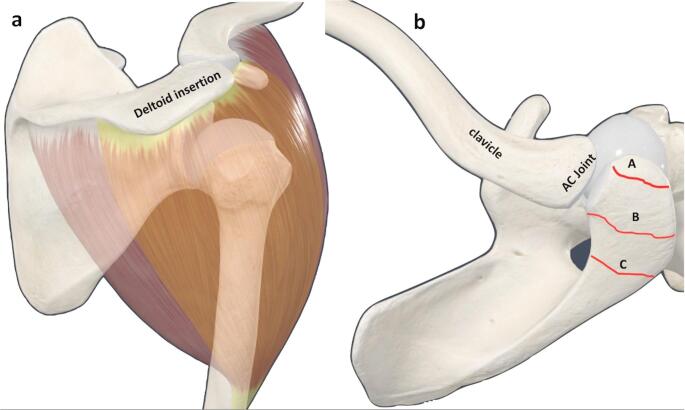
**(a)** Deltoid insertion in the acromion process. **(b)** The types of os acromiale: (A) the space between the os preacromiale and the acromion; (B) the space between the os meso acromiale and the acromion and (C) the space between the os meta-acromiale and the acromion

On the other hand, os acromiale represents an unfused accessory centre of ossification of the acromion of the scapula that normally occurs between adolescence and early adulthood and is estimated to occur in up to 15% of the population [4]. The types of os acromiale are determined according to the unfused segment nonunion (Fig. 1) [5, 6].

We know that the deltoid, although it works as a functional unit, can be divided into three anatomic heads: anterior, middle, and posterior deltoid. A significant portion of the middle deltoid inserts into the acromial process (Fig. 1) and we know that the deltoid under a RTSA is over tensioned because of the previous mentioned distalization, so it would be reasonable to think that a preoperative os acromiale, after the implantation of a RTSA, could potentially be displaced inferiorly due to the excessive tension on the deltoid, caused by its elongation. This, in turn, would alter the lever arm of the deltoid with the consequent functional impairment and higher complication rates related with the loss of deltoid tension such as dislocation.

There is limited published information on the effect of acromial disease on outcomes after RTSA, particularly in patients with an os acromiale [7–12]. Moreover, the existing data is inconclusive in several aspects.

Therefore, the hypothesis of the present study is that the implantation of a RTSA in patients with an os acromiale would cause displacement of the os acromiale, altering the lever arm of the deltoid and resulting in functional impairments and a higher number of complications, such as dislocation.

## Materials and methods

### Study design

After obtaining approval from our Institution Review Board (protocol code C.I. 24/191-E), all patients who had undergone RTSA for arthropathy, primary osteoarthritis or irreparable rotator cuff tear at our institute between January 2004 and December 2021 were identified. A screening for the presence of an os acromiale reviewing preoperative imaging (xray, MRI, CT) was performed. Exclusion criteria included patients who underwent RTSA for proximal humerus fractures, revision surgeries, severe glenoid defects requiring augmentation or fracture sequelae, follow-up of less than two years, and physical disabilities that prevented understanding of functional assessment scales.

Among 432 patients who received a RTSA during the study period, 221 were treated for the aforementioned indications. After applying the exclusion criteria, 12 shoulders from 11 patients with an os acromiale (cases) were paired with 24 shoulders who underwent RTSA during the same period but did not have an os acromiale (controls). The matching criteria included sex, age (closest after applying the other criteria), side and implant type. Three patients were excluded after matching due to follow-up lower than 24 months.

### Surgical procedure

All surgeries were carried out by one of three senior shoulder surgeons (YL, CG-F, and FM), with two of the three surgeons present at each procedure. There was no deviation from standard operative protocols previously described [13], given the presence of an os acromiale. The deltopectoral approach was used in 24 cases and the anterosuperior approach in 12. The subscapularis tendon was reattached with a transosseous suture in all the cases. Implant design with a medialized and distalized centre of rotation (COR) included the Delta Xtend RTSA (DePuy-Johnson & Johnson, Warsaw, IN, USA) and Lima SMR (Lima LTO, San Daniele del Friuli, Italy). The baseplate was positioned in alignment with the inferior edge of the glenoid and rotated so that the superior hole was aligned with the base of the coracoid. Glenoid baseplates were not cemented and were secured with four peripheral locking screws for Delta Xtend or two for Lima SMR. Postoperatively, patients were immobilized in slings and began passive range of motion exercises 24–48 h after surgery. They gradually progressed to increased functional activity and started strengthening atsix weeks.

### Functional and radiological assessment

Pre and postoperative functional outcomes were measured with the Constant scale (CS) [14], range of movement (ROM), Visual Analogue Scale (VAS), ASES and Quick Dash scales. Clinical evaluation was performed by 2 independent surgeons who were not involved in the original surgery (RH, EA). Complications and revision surgeries were also analyzed.

Radiographic assessment was performed by the same independent surgeons that performed functional assessment and included preoperative, immediate postoperative, and final follow-up radiographs to evaluate: (1) the type of os acromiale, classified as preacromion (PA), mesoacromion (MSA), meta-acromion (MTA), and basiacromion (BA) (Fig. 1) (2) The acromiohumeral distance: measured from the most lateral end of the acromion to the top of the greater tuberosity (3) Inferior tilt: evaluated on preoperative and postoperative X-rays to detect any migration or tilt of the unfused segment of the os acromiale [1] (4) Radiolucent lines suggesting loosening were noted [15] (5) Scapular notching was classified according to the Sirveaux classification [16].

### Statistical analysis

Qualitative variables are presented with their frequency distribution and percentages. For continuous variables, the mean and standard deviation (SD) are reported if the data follow a normal distribution; otherwise, the median and interquartile range (IQR) are reported. Demographic and baseline survey data and the radiologic data comparisons between the study and matched control groups were conducted using Chi-square or Fisher exact test for categorical variables and Mann-Whitney U test for continuous variables. Differences in functional results between presurgery and final follow-up in each group were assessed using Wilcoxon test and for comparison between the groups Mann-Whitney U test was used. Statistical significance was set at *p* <.05 for all tests, which were performed using SPSS version 26.0 (IBM, Armonk, NY, USA).

A post hoc power analysis was performed. To detect the minimum clinically important difference (MCID) of the Constant score for shoulder arthroplasty (improvement of 8) [17], assuming a standard deviation of 10 points and a 2:1 ratio of controls to study patients, the number of study patients (= 12) was sufficient to obtain 80% power for α = 0.05.

## Results

### Patients

**Fig. 2:**
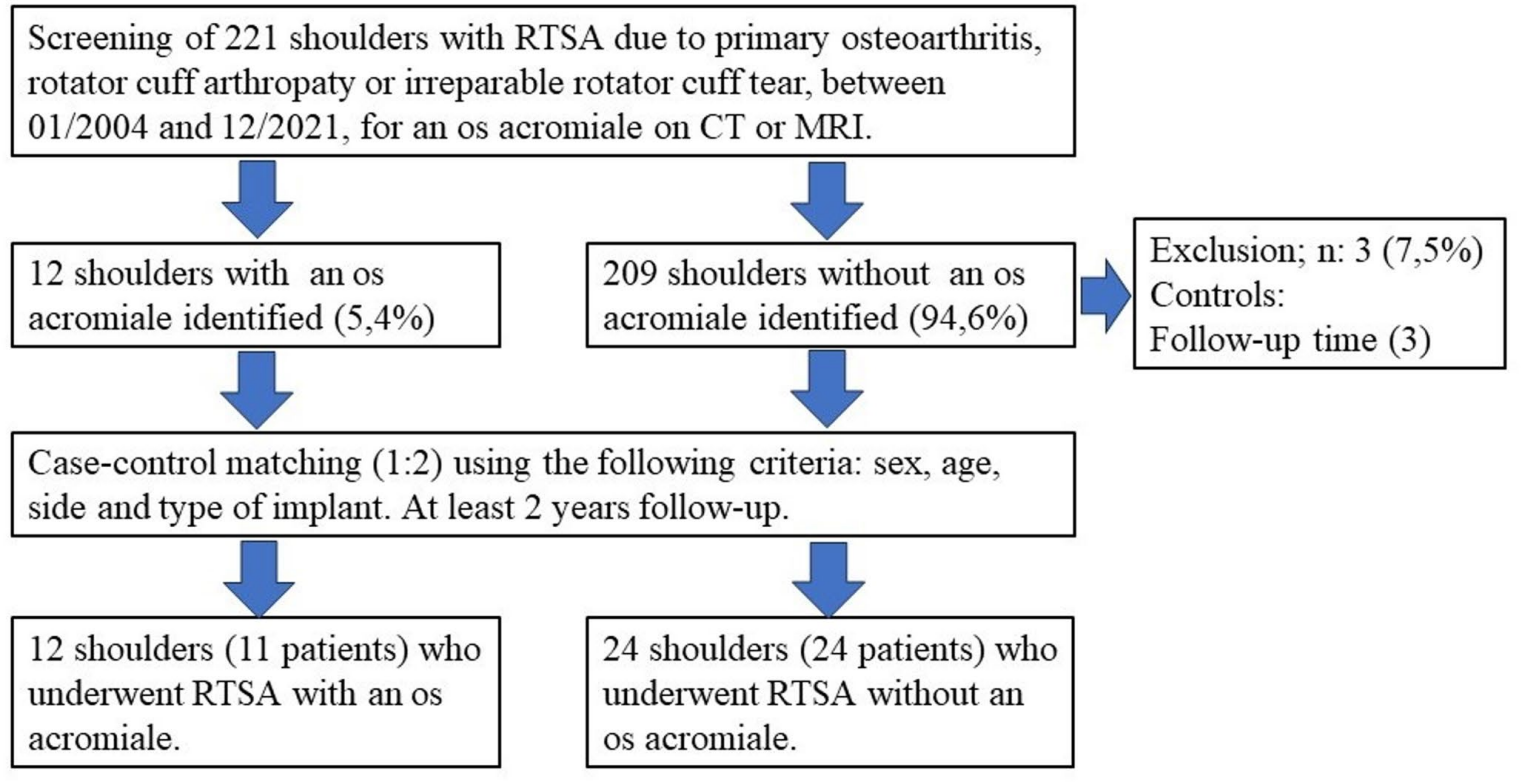
Flowchart of the study

A total of 12 shoulders with an os acromiale (cases: OA) and 24 shoulders without an os acromiale (controls: NOOA) were available after a mean follow-up of 47.2 and 56.1 months respectively, with data from all of them available at a minimum of two years follow-up (Fig. 2).

There were no significant differences between the OA and the NOOA groups in demographic and surgery baseline characteristics (Table 1).

**Table 1:** Baseline patient and surgery characteristics

	OA (*n*:12)	NOOA (*n*:24)	*p*
Demographics			
Age, mean: years (SD/ range)	73.5 ± 4.7 (66–81)	75.4 ± 4.1 (67–82)	0.238
Sex, female (n, %)	10 (83.3%)	20 (83.3%)	1
Side, right (n, %)	9 (75%)	18 (75%)	1
Dominant side (n, %)	8 (66.7%)	21 (87.5%)	0.190
Body mass index: kg/m2 (SD, range)	25.6 ± 2.6 (23–32)	28.6 ± 8.6 (22–44)	0.967
Charlson Index of comorbidities			0.611
1 (n, %)		1 (4.2%)	
2 (n, %)		4 (16.7%	
3 (n, %)	6 (50%)	8 (33.3%)	
4 (n, %)	3 (25%)	5 (20.8%)	
5 (n, %)	2 (16.7%)	3 (12.5%)	
6 (n, %)		2 (8.3%)	
7 (n, %)	1 (8.3%)	1 (4.2%)	
ASA Classification			0.278
1 (n, %)		4 (16.7%)	
2 (n, %)	12 (100%)	20 (83.3%)	
Indication for surgery			0.930
Rotator cuff arthropathy (n, %)	9 (75%)	17 (70.8%)	
Irreparable rotator cuff tear (n, %)	1 (8.3%)	3 (12.5%)	
Primary osteoarthritis (n, %)	2 (16.7%)	4 (16.7%)	
Surgery and implant characteristics			
Previous surgery (arthroscopic tendon repair) (%)	1 (8.3%)	3 (12.5%)	0.620
Approach			1
Deltopectoral (n, %)	8 (66.7%)	16 (66.7%)	
Anterosuperior (n, %)	4 (33.3%)	8 (33.3%)	
Type of implant			1
Lima SMR (n, %)	6 (50%)	12 (50%)	
Delta Xtend (n, %)	6 (50%)	12 (50%)	
Cemented (n, %)	2 (16.7%)	5 (20.8%)	1
Time of follow-up, months (SD, range)	47.2 ± 25 (24–120)	56.1 ± 30 (24–120)	0.491

OA: Os acromiale NOOA: No os acromiale SD: Standard deviation ASA: American Society of Anesthesiologists

### Functional and radiographic assessment

At the end of the follow-up, both cases and controls showed a significant increase in joint range of motion in forward elevation and abduction. However, no significant differences were observed between the groups regarding the improvement experienced (Table 2).

Regarding the functional scales and VAS, both groups showed significant improvement at the final follow-up compared to their preoperative situation. However, the improvement in ASES and Constant scores at the final follow-up was significantly greater in the NOOA group compared to the OA group. When the four variables of the Constant score (pain, activities of daily living, range of motion, and strength) were analyzed at the final follow-up, only the strength component showed statistically significant differences between the two groups, with a mean score of 5.8 ± 4.42 for the os acromiale group versus 7.74 ± 5.72 for the NOOA group (*p* =.030).

**Fig. 3:**
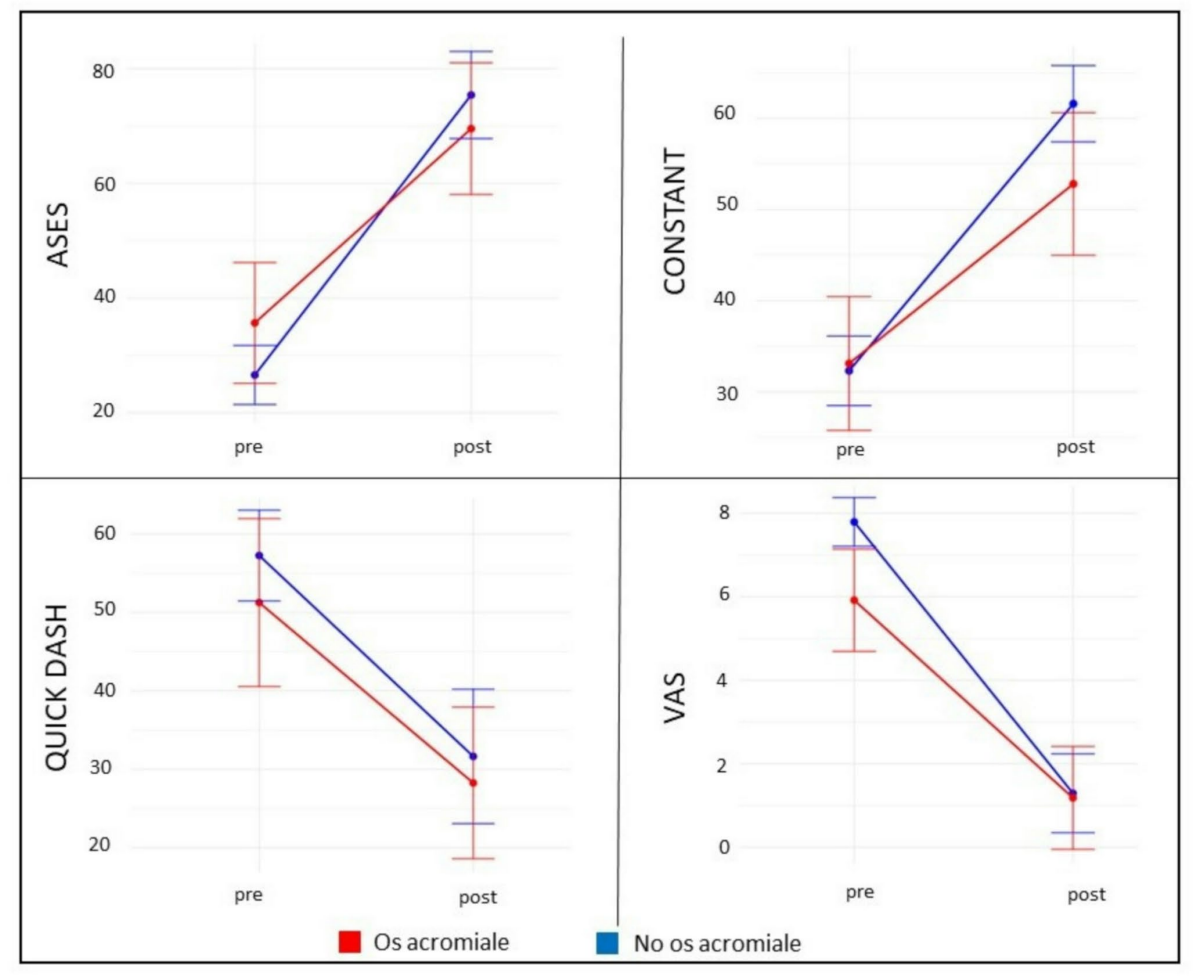
Functional outcome in scales pre: presurgery, post: at the end of follow-up dif: Difference between the presurgery and the end of follow-up measures

The pre-surgery VAS was higher in the NOOA group, which experienced greater improvement than the OA group, resulting in a similar score at the end of follow-up (Table 2; Fig. 3).

**Table 2:** Functional evaluation

Function	Time of assessment	OA (12)	NOOA (24)	*p*
Forward elevation (º)	Presurgery	85º (50–120)	90º (50–110)	0.736
	End follow-up	140º (100–150)	135º (110–155)	0.747
	Difference	50º (15–55)	60º (20–80)	0.138
Abduction (º)	Presurgery	70º (40–95)	80º (40–90)	0.919
	End follow-up	140º (100–160)	130º (90–160)	0.603
	Difference	60º (30–70)	60º (10–80)	0.775
External rotation (º)	Presurgery	10º (5–15)	8º (0–25)	0.784
	End follow-up	10º (10–20)	15º (13–16)	0.727
	Difference	0º (5–15)	0º (10–17)	0.900
ASES	Presurgery	36 (25-47.7)	25 (18.5–30)	0.041*
	End follow-up	75 (53–85)	80 (69–87)	0.224
	Difference	28 (18–52)	54 (35.2–60.7)	0.017*
CONSTANT	Presurgery	35.8 (24.7–41)	34.5 (28.3–39)	0.777
	End follow-up	56 (50–61)	63.7 (54.5–70)	0.039*
	Difference	20.2 (10.1–24)	30.9 (21.9–38.1)	0.012*
Quick-Dash	Presurgery	42.9 (38.6–63.6)	59.1 (46.6–65.9)	0.178
	End follow-up	21.3 (15.9–38.6)	28.4 (16.5–47.1)	0.709
	Difference	-19.6 (-27.2–18.1)	-27.2 (-34.1–15.9)	0.220
VAS	Presurgery	5.5 (5–7)	8 (7–9)	0.005*
	End follow-up	0 (0–1)	0 (0-1.7)	1
	Difference	-5 (-5–4)	-7 (-8–5.2)	0.007*

OA: Os acromiale NOOA: No os acromiale VAS: Visual analogue scale. IQR: Interquartile range. All the results are expressed as the median and Interquartile range

Regarding the radiological measurements, the acromiohumeral distance and the inferior tilt increased significantly from the initial to the final observation.

Regarding the radiological measurements, the acromiohumeral distance and the inferior tilt increased significantly from the initial to the final observation in both groups (Table 3).

**Table 3:** Radiologic outcomes

Radiologic assessment	Time of assessment	Type	OA (*n*:12)	NOOA (*n*:24)	*p*
Os acromiale		PA	1		
		MSA	11		
Acromiohumeral distance median mm (IQR)	Presurgery		5 (1–12)	5 (4–11)	0.946
	Final assessment		26 (16–28)	27 (20–33)	0.154
	Difference		20 (11–21)	19 (13–26)	0.581
Inferior tilt. median grades (IQR)	Presurgery		22 (16–24)	13 (8–17)	0.006*
	Final assessment		28 (17–43)	14 (10–25)	0.018*
	Difference		5 (0–21)	2 (-1-8)	0.294
Radiolucence, n (%)	Final assessment		1 (8.3%)	2 (8.3%)	1
Notching, n (%)	Final assessment	Type I	1 (8.3%)	2 (8.3%)	1
		Type II	1 (8.3%)	2 (8.3%)	

OA: Os acromiale NOOA: No os acromiale PA: Preacromion, MSA: Mesoacromion, IQR: Interquartile range

### Postoperative complications and revision surgery

**Fig. 4:**
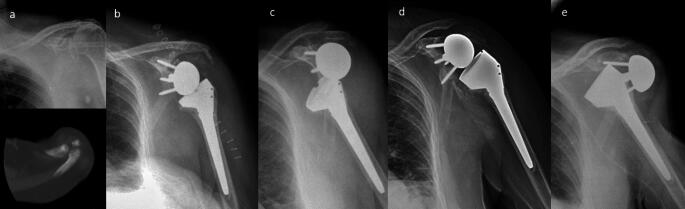
A 72-year-old woman with recurrent prosthetic instability (**a**) X-ray and axial CT plane showing rotator cuff arthropathy with os acromiale (preacromion). (**b**) Immediate postoperative radiographic control. (**c**) Prosthetic dislocation after two years. (**d**) Radiographic control after revision surgery. (**e**) Third dislocation, no further revision surgery performed

**Fig. 5:**
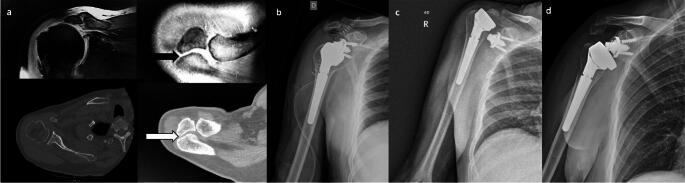
A 66-year-old patient with prosthetic instability (**a**) MRI: Rotator cuff arthropathy with os acromiale (mesoacromion), coronal and axial plane. CT: Axial plane on glenohumeral joint and os acromiale. (**b**) Immediate postoperative radiographic control. (**c**) Prosthetic dislocation at 6 weeks. (**d**) Radiographic control after revision surgery

No prosthesis infections or radiolucent lines were observed around the humeral and glenoid components at the final radiographic follow-up in either group. Scapular notching was noted in two cases within the os acromiale group (16.6%); one classified as grade 1, and the other as grade 2. One patient in the NOOA group suffered a periprosthetic fracture after a fall, which did not require surgical treatment. Additionally, there were no dislocations at the last follow-up in the control group, while two cases were reported in the os acromiale group. A 72-year-old woman had a dislocation two years post-RTSA. After two revision surgeries with component changes (polyethylene liner and glenosphere), she had a third dislocation without trauma (Fig. 4). She opted against further surgery due to the absence of pain and other health issues. Cultures from previous surgeries were negative for infection. A 66-year-old man experienced a dislocation six weeks post-RTSA without trauma (Fig. 5). Revision surgery involved increasing the glenosphere size and using a retentive insert, with no intervention on the os acromiale.

## Discussion

The prevalence of os acromiale varies from 1.9 to 15% depending on the population studied [18]. Some authors suggest that os acromiale might contribute to the development of rotator cuff tears and, subsequently, cuff tear arthropathy, given its higher incidence in patients with these conditions compared to the general population [10]. However, the frequency found in the present study (5.4%) was not higher than what has been reported in the general population [19, 20].

The relationship between os acromiale and the functional outcomes and complications following RTSA has been poorly investigated [7–12].

This study identified significant differences in clinical outcomes (ASES, Constant, and VAS) after RTSA between patients with os acromiale and matched controls without it. Patients with os acromiale not only demonstrated worse functional outcomes but also a higher rate of complications. These findings have significant clinical implications, emphasizing the importance for surgeons to increase attention to intraoperative prosthetic stability. This contrasts with previous studies where the presence of an os acromiale did not result in worse functional outcomes or an increased complication rate.

Some previous studies do not include a comparison group and only analyze results from patients with os acromiale. However, the issue is not whether a patient with os acromiale and RTSA improves—the existing literature agrees they do. The debate should focus on whether patients with os acromiale experience the same level of improvement as those without it. In the present study, both groups achieved similar results in ROM, but not in strength. The os acromiale group had significantly lower strength scores compared to the non-os acromiale group, which was the main reason for the differences found in the Constant score. Walch et al. [11] suggested that while the anterior and posterior portions of the deltoid, which insert on the intact scapular spine and clavicle, can compensate for the compromised lateral deltoid in os acromiale, this compensation might maintain range of motion but is insufficient for restoring strength.

Although the NOOA group started with a higher VAS pain score, the final result was the same for both groups. The greater pain relief in the NOOA group likely explains their higher improvement on the ASES scale.

In terms of radiological outcomes, the design of reverse shoulder arthroplasty, which enhances the lever arm, lowers the humerus, and increases deltoid muscle fiber recruitment, places greater stress on the acromion, potentially leading to postoperative displacement of the os acromiale. In the present study, a reduced acromiohumeral distance after surgery in patients with os acromiale was observed, along with a more pronounced “inferior tilt” of the acromion compared to controls. This displacement of the os acromiale following reverse prosthesis implantation has been reported by other authors as well, without finding any relation between the amount of displacement and the final outcome [7, 9, 11, 21].

Patients with os acromiale exhibited higher complication rates in our study, particularly instability (16% vs. 0%). Previous studies generally have not reported increased complication rates, except for occasional postoperative pain that typically subsides over time [7].

The higher rate of instability observed in the present study could be related to the type of implant used, which involves medialization of the centre of rotation. Although not all studies specify the type of implant used, most involve RTSA with a lateralized onlay-type humeral component [7, 9–11].

As previously explained, Walch et al. [11] attribute the minimal functional impact of middle deltoid insufficiency caused by os acromiale to the integrity of the anterior and posterior portions. However, the Grammont-style prosthesis leads posterior deltoid tension loss due to medialization. This, combined with the loss of middle deltoid tension due to os acromiale, could explain the higher rate of instability. Conversely, a lateralized humeral design is more efficient at increasing the moment arms for the teres minor, infraspinatus, and posterior deltoid muscles [22].

Initially, some authors recommended aggressive surgical treatment for os acromiale, such as osteosynthesis during RTSA to preserve the deltoid insertion and enhance arm elevation [23]. However, osteosynthesis appears challenging, and additionally consistent outcomes and union rates have not been reliably reported [24]. Moreover, favourable outcomes without osteosynthesis [11] prompt questioning effectiveness of this approach.

In our view, os acromiale does not represent a contraindication for surgery. However, unlike other authors who believe no modifications to the surgical technique are necessary in cases of os acromiale [9], we recommend implementing specific modifications to prevent potential instability, especially when using Grammont-style medialized implants. These include consistently repairing the subscapularis tendon, using larger glenospheres and preserving the coracoacromial ligament (CAL). We believe that preserving CAL may be useful because of the established relationship between preserving the CAL and reducing acromial stress [25]. Finally, we should consider the use of implants with lateralization of the humeral component, given the previously mentioned effects of such designs on the posterior deltoid and the greater risk of destabilizing a stable os acromiale when using medialized implants (Grammont design) that require more elongation to achieve proper deltoid tension.

The present study has significant limitations inherent to its retrospective design. The sample size was relatively small, drawn from a registry database, and we could not add more patients to increase statistical power. However, a post hoc power analysis showed adequate power to detect the minimal clinically important difference in the Constant score between groups. Furthermore, the study population included various indications for RTSA and two prosthesis types, although both shared the Grammont design philosophy. This may introduce unwanted heterogeneity in outcomes. To minimize bias, we used appropriate matching (indications and implant type) between groups. Additionally, no specific physical exam maneuvers were performed pre- or postoperatively to assess acromial pain. Lastly, this study only reports medium-term follow-up, limiting extrapolation of long-term outcomes for RTSA in patients with os acromiale.

## Conclusions

Patients with os acromiale treated with RTSA show improvement in their preoperative clinical condition, but the degree of improvement is less than that observed in patients without os acromiale. While both groups achieve similar joint balance, the poorer outcomes on functional evaluation scales in the os acromiale group are primarily due to reduced strength and less pain relief following RTSA implantation. Additionally, patients with os acromiale who receive an implant that medializes the centre of rotation experience a higher rate of instability compared to those without os acromiale.

## Data Availability

The data that support the findings of this study are available from Clínico San Carlos Hospital, Madrid, and so are not publicly available. The data are, however, available from the authors upon reasonable request and with the permission of Clínico San Carlos Hospital.
